# Integrated miRNA and mRNA expression profiling of the inflammatory breast cancer subtype

**DOI:** 10.1038/sj.bjc.6605787

**Published:** 2010-07-27

**Authors:** I Van der Auwera, R Limame, P van Dam, P B Vermeulen, L Y Dirix, S J Van Laere

**Affiliations:** 1 Translational Cancer Research Group (Laboratory of Pathology, University of Antwerp/University Hospital Antwerp; Oncology Centre, Sint-Augustinus), Oosterveldlaan 24, B-2610 Antwerp, Belgium

**Keywords:** microRNA, inflammatory breast cancer, dicer, ago2, mRNA

## Abstract

**Background:**

MicroRNAs (miRNAs) are key regulators of gene expression. In this study, we explored whether altered miRNA expression has a prominent role in defining the inflammatory breast cancer (IBC) phenotype.

**Methods:**

We used quantitative PCR technology to evaluate the expression of 384 miRNAs in 20 IBC and 50 non-IBC samples. To gain understanding on the biological functions deregulated by aberrant miRNA expression, we looked for direct miRNA targets by performing pair-wise correlation coefficient analysis on expression levels of 10 962 messenger RNAs (mRNAs) and by comparing these results with predicted miRNA targets from TargetScan5.1.

**Results:**

We identified 13 miRNAs for which expression levels were able to correctly predict the nature of the sample analysed (IBC *vs* non-IBC). For these miRNAs, we detected a total of 17 295 correlated miRNA–mRNA pairs, of which 7012 and 10 283 pairs showed negative and positive correlations, respectively. For four miRNAs (miR-29a, miR-30b, miR-342-3p and miR-520a-5p), correlated genes were concordant with predicted targets. A gene set enrichment analysis on these genes demonstrated significant enrichment in biological processes related to cell proliferation and signal transduction.

**Conclusions:**

This study represents, to the best of our knowledge, the first integrated analysis of miRNA and mRNA expression in IBC. We identified a set of 13 miRNAs of which expression differed between IBC and non-IBC, making these miRNAs candidate markers for the IBC subtype.

MicroRNAs (miRNAs) are a class of non-coding RNAs able to regulate gene expression at the post-transcriptional level, by binding to the 3′ untranslated region of target messenger RNAs (mRNAs) through partial sequence homology, and causing a block of translation and/or mRNA degradation (He and Hannon, 2004). At the time of writing of this paper, 721 human miRNA genes had been described (http://microrna.sanger.ac.uk/sequences) and it has been estimated that each of the miRNAs targets about 100 different mRNA molecules (Brennecke *et al*, 2005; Lewis *et al*, 2005; Lim *et al*, 2005). MiRNAs have important roles in essential processes, such as differentiation, cell growth, stress response and cell death (Miska, 2005; Zamore and Haley, 2005). Accordingly, altered miRNA expression is likely to contribute to human disease, including cancer. In fact, the spectrum of miRNAs expressed in solid cancers is very different from that of normal cells and the predicted targets for the differentially expressed miRNAs are significantly enriched for protein-coding tumour suppressors and oncogenes (Lu *et al*, 2005; Volinia *et al*, 2006).

Focussing on breast cancer, levels of specific miRNAs differ between malignant and normal breast tissue and are able to classify tumours according to clinicopathological variables, such as proliferation index, steroid hormone receptor and Her2/neu status, nodal status and tumour stage (Iorio *et al*, 2005; Mattie *et al*, 2006; Lowery *et al*, 2008). Furthermore, miRNAs are differentially expressed between molecular breast cancer subtypes, including luminal A, luminal B, basal-like and Her2+ (Blenkiron *et al*, 2007). This highlights the potential of miRNA signatures as new prognostic indicators that may contribute to the improved selection of patients for adjuvant therapy. Indeed, Foekens *et al* (2008) recently linked four miRNAs (*miR-7*, *miR-128a*, *miR-210* and *miR-516-3p*) to a shorter time to distant metastasis in oestrogen receptor (ER)-positive, lymph node-negative breast cancer.

In this study, we explored whether inflammatory breast cancer (IBC) is associated with a specific miRNA signature. Although this form of breast cancer has traditionally been understudied, there are several reasons for focusing on IBC. First, IBC is arguably the deadliest form of breast cancer, with 5- and 10-year disease-free survival rates of less than 35 and 20%, respectively (Jaiyesimi *et al*, 1992), highlighting the need for accurate prognostic and predictive markers and the development of new molecular targeted therapies. Second, most patients with IBC have lymph node involvement at time of diagnosis and one-third of patients present with distant metastasis (Jaiyesimi *et al*, 1992), which makes IBC an interesting model for identifying the forces driving breast cancer aggressiveness in general. Indeed, several studies comparing IBC to non-IBC have demonstrated a specific mRNA expression signature in IBC that is significantly enriched for genes involved in cell motility, inflammation, immune response and stem cell biology (Bertucci *et al*, 2004, 2005; Van Laere *et al*, 2005, 2006, 2007). Furthermore, we recently reported that the identified IBC gene signature was associated with dismal tumour characteristics in non-IBC, such as high tumour grade, absence of ER expression and presence of Her2/neu expression, and independently predicted relapse-free survival in non-IBC (Van Laere *et al*, 2008). Finally, accurate and rapid diagnosis of IBC remains problematic due to the lack of uniformity in criteria for IBC diagnosis and new diagnostic markers are therefore needed.

## Materials and Methods

### Patients’ samples

Tumour samples were retrieved from the tissue bank of the General Hospital Sint-Augustinus (Antwerp, Belgium). Clinical and pathological data are stored in a database in accordance with hospital privacy rules. Specimens were brought to the pathologists immediately after resection and part of the tissue was placed in liquid nitrogen and subsequently stored at −180°C. A total of 20 patients with IBC and 50 patients with non-IBC were included in this study. Inflammatory breast cancer was diagnosed according to the criteria mentioned in the AJCC (American Joint Committee on Cancer)-TNM staging system (Singletary *et al*, 2002). All patients with IBC showed diffuse enlargement of the involved breast of sudden onset. There was erythema and oedema of the skin involving more than one-third of the breast. The presence of dermal lymphatic invasion as an isolated observation was not sufficient for the diagnosis of IBC and was not necessary for the diagnosis either. The non-IBC control group was matched for histological tumour grade, ER expression and human epidermal growth factor receptor 2 (HER2) amplification but not for tumour stage to identify true determinants of IBC. Tumour characteristics are provided in Table 1.

### Total RNA isolation, complementary DNA synthesis and miRNA quantification

After tissue disruption, total RNA was extracted by using the *mir*Vana miRNA Isolation Kit (Ambion, Austin, TX, USA) according to the manufacturer's instructions for total RNA isolation. Purified total RNA was quantified by using a NanoDrop ND1000 (NanoDrop Technologies, Wilmington, DE, USA). Total RNA (100 ng) was then converted to complementary DNA by priming with a mixture of stem-looped RT primers (Megaplex RT Primers, Human Pool A, Applied Biosystems, Foster City, CA, USA) in combination with the TaqMan MicroRNA Reverse Transcription Kit (Applied Biosystems), allowing simultaneous transcription of 377 unique miRNAs and six endogenous controls. Briefly, 3 *μ*l of total RNA was supplemented with RT primer mix (10 ×), dNTPs with dTTP (100 mM), Multiscribe Reverse Transcriptase (50 U *μ*l^−1^), RT buffer (10 ×), MgCl_2_ (25 mM) and RNase inhibitor (20 U *μ*l^−1^) in a total reaction volume of 7.5 *μ*l. Thermal-cycling conditions were as follows: 40 cycles of 16°C for 2 min, 42°C for 1 min and 50°C for 1 s, followed by a reverse transcriptase inactivation at 85°C for 5 min. The Megaplex RT product (2.5 *μ*l) was pre-amplified using the TaqMan PreAmp Master Mix (Applied Biosystems) and PreAmp Primers, Human Pool A (Applied Biosystems) in a 25-*μ*l PCR reaction. Thermal-cycling conditions were as follows: 95°C for 10 min, 55°C for 2 min and 75°C for 2 min, followed by 12 cycles of 95°C for 15 s and 60°C for 4 min. MiRNA quantification was performed with the TaqMan Human MicroRNA A Array Set v2.0 (Applied Biosystems), which contains 384 TaqMan miRNA assays. The PreAmp product was diluted four-fold. Each of the eight wells was loaded with 100 *μ*l of PCR reaction mix, containing 50 *μ*l of TaqMan Universal PCR Master Mix, no AmpErase uracil *N*-glycosylase (UNG) (Applied Biosystems), 1 *μ*l of diluted PreAmp product and 49 *μ*l of nuclease-free water. Thermal-cycling conditions were as follows: 94.5°C for 10 min, followed by 40 cycles of 97°C for 30 s and 59.7°C for 1 min. All PCR reactions were performed on a 7900HT Fast Real-Time PCR System (Applied Biosystems). The PCR replicates were measured for four samples.

### Statistics and bioinformatics

To reduce technical variation, all miRNAs with a *C*_t_ (threshold cycle) detection cut-off of less than 35 PCR cycles in at least 25% of samples were filtered, resulting in a list of 322 informative miRNAs. Next, we performed a between-sample normalisation by median-centring the distribution of miRNA expression levels for each sample (Mestdagh *et al*, 2009). Relative miRNA expression levels were calculated using the Δ*C*_t_ method (Livak and Schmittgen, 2001).

To identify miRNA expression patterns, we performed an unsupervised hierarchical complete linkage cluster analysis using Euclidean distance as dissimilarity metric. The number of robust sample clusters was determined with the silhouette algorithm. Next, we investigated the association of the sample clustering result with clinicopathological variables using a Pearson *χ*^2^ test and a Goeman global test (Goeman *et al*, 2004).

To identify individual miRNAs associated with IBC, we first selected all miRNA that were significantly over- or underexpressed in IBC when compared with non-IBC using a non-parametric test. Then, each of these miRNAs was subjected to a multivariate regression analysis with N status, M status, tumour stage and HER2 amplification as covariates. In this way, we could indentify miRNAs that are specifically related to IBC and not to differences in clinicopathological variables between IBC and non-IBC known to also influence miRNA expression in breast cancer.

To investigate the biological relevance of the identified miRNAs, we adopted the strategy recently described by Wang *et al* (2009). First, for each of the miRNAs that were independently associated with IBC, we identified a set of putative target genes by Spearman correlation analysis, taking into account both positive and negative correlations. This analysis was performed on a subgroup of 44 samples (20 IBC and 24 non-IBC samples) for which Affymetrix HGU133 plus 2.0 gene expression profiles were available (Van Laere *et al*, 2007). Next, to identify the true miRNA targets, we compared the lists of putative miRNA targets with the lists of miRNA targets defined by TargetScan5.1 (http://www.targetscan.org) using a hypergeometric gene set enrichment analysis. Those miRNAs for which the miRNA targets defined by TargetScan5.1 were significantly enriched for correlation-defined miRNA targets were subjected to further analysis. In addition, all further analyses were performed only on the common genes between the two miRNA targets sets. These genes were analysed for enriched Gene Ontology (GO) and Kyoto Encyclopedia of Genes and Genomes (KEGG) categories through hypergeometric gene set enrichment analysis.

To test whether the identified miRNAs are prognostically relevant, we downloaded six publicly available gene expression data sets from the National Center for Biotechnology Information website (GSE7390, GSE9195, GSE1456, GSE11121, GSE2034 and GSE4922; http://www.ncbi.nlm.nih.gov). In addition, the data set described by van de Vijver *et al* (2002) was retrieved from the Rosetta website (http://www.rii.com). In total, we analysed mRNA expression data from 1504 breast cancer samples. For each sample, we calculated a score, proportional to the level of expression of a selected miRNA, by subtracting the average of the negatively correlated miRNA targets from the average of the positively correlated miRNA targets. These scores were standardised (median=0 and s.d.=1) to allow for comparisons across different data sets and analysed using a Cox proportional hazards model in both a univariate and a multivariate setting. The outcome variable was distant metastases-free survival (DMFS, *N*=1059), overall survival (OS, *N*=652) or relapse-free survival (RFS, *N*=1145) where possible.

Finally, within the subset of 44 samples for which Affymetrix HGU133 plus 2.0 gene expression profiles were available, we compared the expression of miRNA processing genes between IBC and non-IBC. Furthermore, we analysed the putative regulatory effect of all miRNAs from the TargetScan5.1 database on the gene expression profiles from these 44 samples. We adopted the approach recently published by Cheng *et al* (2009) to calculate a regulatory effect (RE) score by subtracting the average rank of the miRNA targets from the average rank from the non-miRNA targets, with high RE scores denoting a strong effect of the miRNA on the expression of the targets and *vice versa*. However, as this score only takes into account the negatively regulated miRNA targets, we adapted the RE score in a manner that both inhibitory and activating effects were equally weighed. Next, these RE scores were compared between IBC and non-IBC using a non-parametrical test.

All data analyses were performed using Bioconductor in R (http://www.bioconductor.org). Correction for multiple testing was performed using the Benjamini and Hochberg step-up false discovery rate controlling procedure and adjusted *P*-values <0.1 were considered significant.

## Results

### Hierarchical clustering based on miRNA expression profiles

We used quantitative RT-PCR technology in combination with a limited cycle pre-amplification to evaluate the miRNA expression profiles of 20 IBC and 50 non-IBC samples. The TaqMan Human MicroRNA A Array v2.0 contains 384 TaqMan miRNA assays enabling accurate quantification of 377 human miRNAs, six endogenous controls and one negative control. Four breast cancer samples were assayed in duplicate. Pearson correlation coefficients for these duplicates ranged from 0.98 to 0.99, indicating good assay reproducibility.

The accuracy of quantitative RT-PCR experiments is dependent on proper data normalisation. For each individual breast cancer sample, we calculated the mean expression value based on those miRNAs that were expressed according to a *C*_t_ detection cut-off of less than 35 PCR cycles in at least 25% of samples (*N*=322). This value was subsequently used as a normalisation factor to reduce technical variation. This normalisation strategy has been shown to be superior to the use of stable small RNA controls (Mestdagh *et al*, 2009).

**Figure 1:**
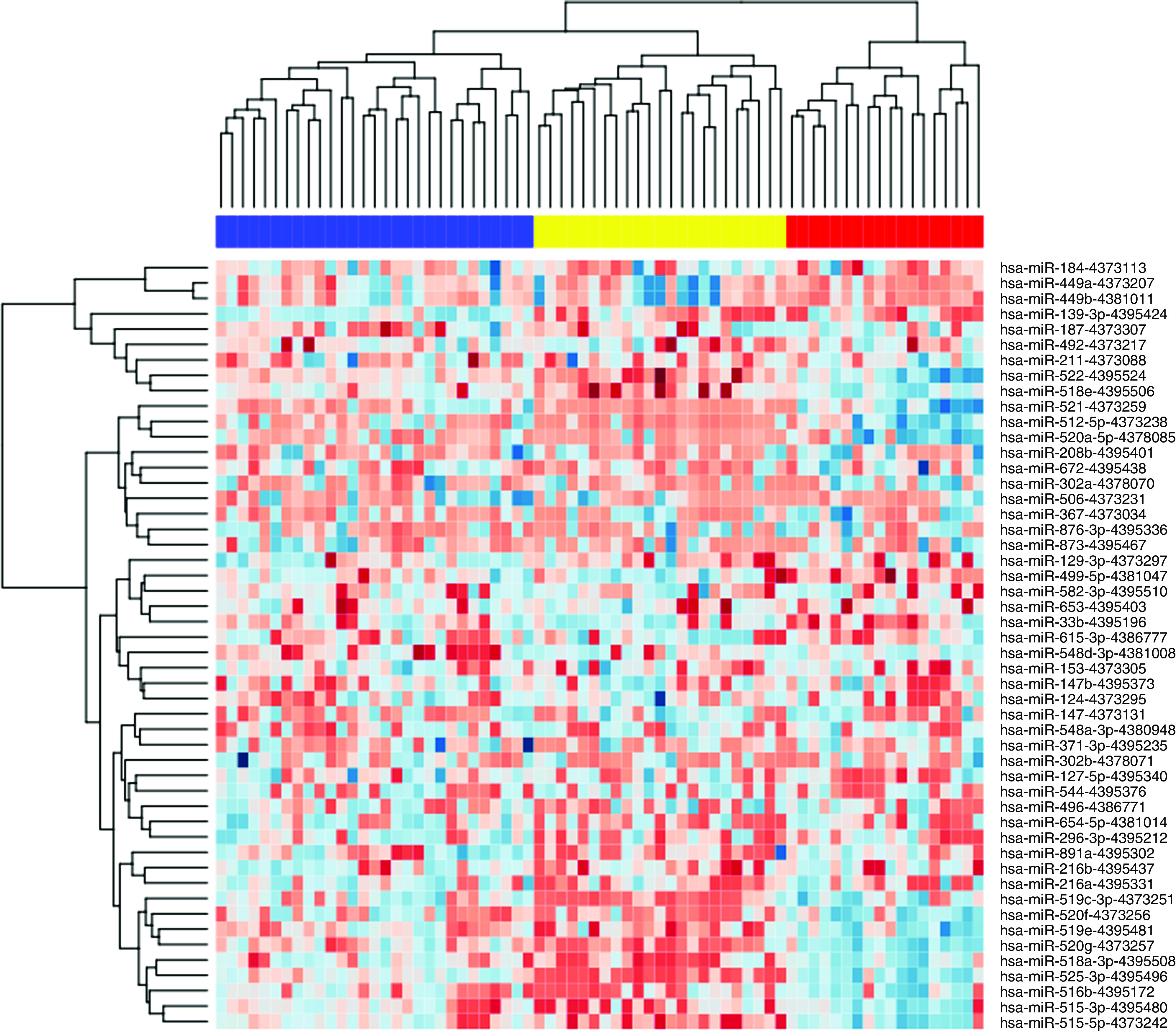
Hierarchical clustering of 20 IBC and 50 non-IBC samples according to the expression pattern of the 50 most varying miRNAs. Expression values for these 50 miRNAs are represented in a matrix format, with rows indicating miRNAs and columns indicating samples. High expression values are colour-coded red and low expression values are colour-coded blue. Three robust sample clusters were identified, which were significantly associated with ER expression and histological grade. In particular, the combined first two (blue and yellow) sample clusters were enriched for ER^+^ breast tumours (80% of samples) when compared with the third (red) sample cluster (50% of samples) (*P χ*^2^=0.028). Notably, in the first (blue) sample cluster, 20% of samples were poorly differentiated compared with 60% of samples in the second (yellow) sample cluster (*P χ*^2^=0.004), suggestive of a subdivision of ER^+^ breast tumours according to the luminal A and luminal B subtype. No association of sample clustering with the difference between IBC and non-IBC was observed: 30, 25 and 45% of IBC samples grouped together in the first (blue), second (yellow) and third (red) sample cluster, respectively (*P χ*^2^=0.082). (The colour reproduction of this figure is available on the html full text version of the manuscript.)

The clustering of miRNA expression profiles derived from 70 breast cancer samples is shown in Figure 1. The dendrogram was constructed by using the 50 most varying miRNAs among all breast cancer samples (based on the s.d. value of miRNA levels across all samples). We identified three sample clusters with an average silhouette width of 0.08 (*P*<0.05 after permutation testing). A Pearson *χ*^2^ test demonstrated that sample clustering was associated with ER expression (*P*=0.011), histological tumour grade (*P*=0.004), N status (*P*=0.014) and tumour stage (*P*=0.010). No association with sample clustering was observed for T and M status, HER2 amplification or tumour subtype (IBC or non-IBC).

**Figure 2:**
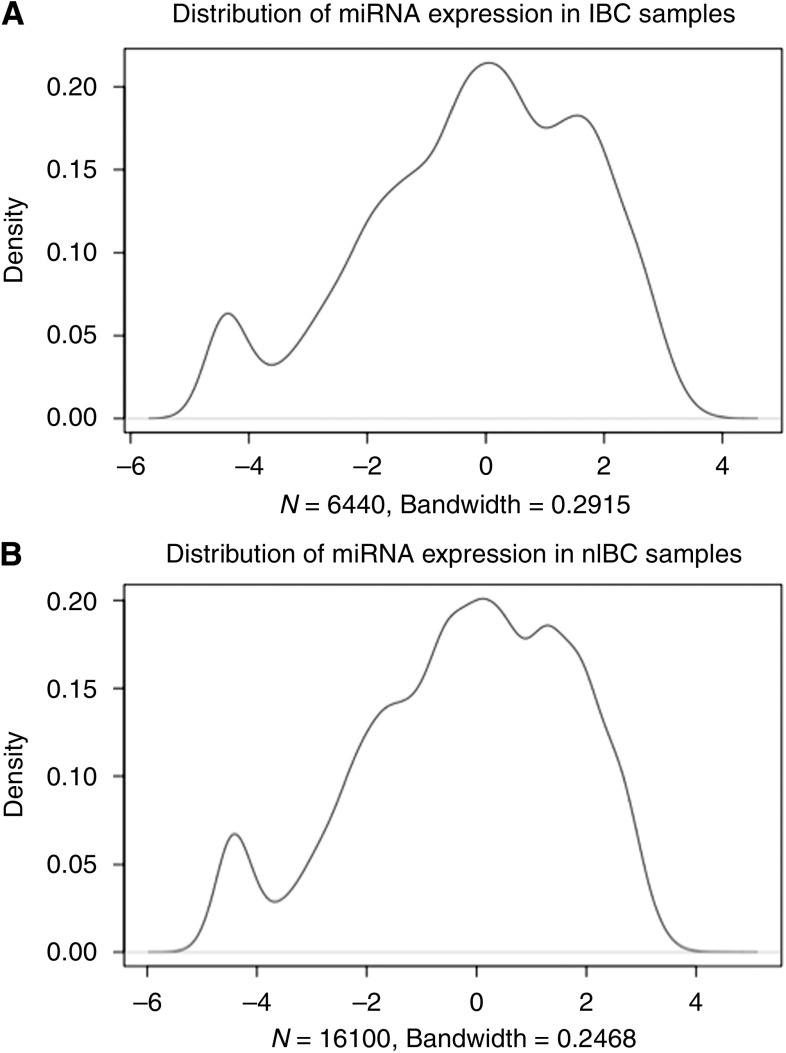
Distribution of miRNA expression values in (**A**) inflammatory breast tumours and (**B**) non-inflammatory breast tumours.

A Goeman global test was performed to look for associations of miRNA expression with various clinicopathological factors (Table 2), for the reduced data set consisting of the 50 most varying miRNAs. Significant associations were observed for ER expression and histological tumour grade, but not for T, N or M status, tumour stage, HER2 amplification or tumour subtype (IBC or non-IBC). Thus, the largest variation in miRNA expression in our set of breast cancer samples was attributable to differences in steroid hormone receptor expression and histological tumour grade and not to differences between IBC and non-IBC. In fact, the overall distributions of miRNA expression values in IBC and non-IBC look very similar (Figure 2).

### Association of individual miRNAs with inflammatory breast cancer

A logistic regression analysis was performed to identify differences in individual miRNA expression levels between IBC and non-IBC samples (Table 3). Few miRNAs were observed to be independently associated with the difference between IBC and non-IBC. Increased expression of miR-335, miR-337-5p, miR-451, miR-486-3p, miR-520a-5p and miR-548d-5p was observed in the IBC subtype, whereas miR-15a, miR-24, miR-29a, miR-30b, miR-320, miR-342-5p and miR-432-3p were significantly downregulated in comparison with non-IBC.

A similar analysis was performed to identify differences in miRNA expression between samples according to T status (low *vs* high), N status (positive *vs* negative), M status, tumour stage (low *vs* high), tumour grade (high *vs* low), ER expression and HER2 amplification. The associations of miRNAs with these clinicopathological factors are also shown in Table 3.

### Prediction of miRNA targets and their involvement in biological processes

Next, we investigated the involvement of miRNA target genes in various biological processes, which may indicate the function of the miRNA, by adopting the strategy recently described by Wang *et al* (2009). First, we performed a correlation coefficient analysis to evaluate potential correlations between expression levels of the 13 miRNAs associated with IBC (miR-335, miR-337-5p, miR-451, miR-486-3p, miR-520a-5p, miR-548d-5p, miR-15a, miR-24, miR-29a, miR-30b, miR-320, miR-342-5p and miR-342-3p) and mRNA expression levels of 10 961 known genes in a reduced set of 44 breast cancer samples, of which 20 were IBC and 24 were non-IBC. Using a false discovery rate of <0.1, we detected significant correlations in 17 295 miRNA–mRNA pairs (Table 4). Of the 17 295 pairs, 7012 pairs and 10 283 pairs showed negative and positive correlations, respectively. For all miRNAs, with the exception of miR-520a-5p and miR-337-5p, we observed more frequently positively correlated miRNA–mRNA pairs than negatively correlated miRNA–mRNA pairs. Subsequently, we performed a gene set enrichment analysis to investigate whether these sets of miRNA-correlated genes are indeed differentially expressed in IBC and non-IBC (Table 4). Significant results were observed for all but one miRNA-correlated gene set. Expression levels of the miR-335/miR-548d-5p/miR-451-correlated gene sets were increased in IBC, whereas expression levels of the miR-15a/miR-24/miR-29a/miR-30b/miR-320/miR-342-5p/miR-342-3p/miR-337-5p/miR-520a-5p-correlated gene sets were decreased in IBC.

To test whether the identified miRNA-correlated genes are direct miRNA targets and not downstream genes of the miRNA targets, we downloaded the predicted miRNA targets from TargetScan5.1 and compared them with the miRNA-correlated genes. The percentages of overlapping genes between the two lists ranged from 0 to 10%. We observed four miRNAs (miR-29a, miR-30b, miR-342-3p and miR-520a-5p) of which targets were significantly enriched among their correlated genes using a false discovery rate of <0.1 (Table 4). The number of overlapping genes was 121 for miR-29a, 140 for miR-30b, 19 for miR-342-3p and 13 for miR-520a-5p (see Table 5 for all target gene symbols).

To functionally classify the direct target genes of miR-29a, miR-30b, miR-342-3p and miR-520a-5p, we performed a gene set enrichment analysis through hypergeometric testing. We focussed on the GO and KEGG gene set collections. For each of the gene sets, we observed various degrees of GO and KEGG term enrichments. The top 10 terms for GO categories or KEGG pathways are listed in Table 6 for each of the four miRNA target gene sets. For miR-29a target genes, the most marked GO terms related to DNA methyltransferase activity. MiR-30b target genes mostly associated with GO terms related to the insulin receptor-signalling pathway. In the miR-342-3p target gene set, cell proliferation-related GO terms were overrepresented. For miR-520a-5p target genes, the most marked GO term was regulation of cell growth by extracellular stimulus.

### Association of miRNA targets with prognosis in breast cancer

**Figure 3:**
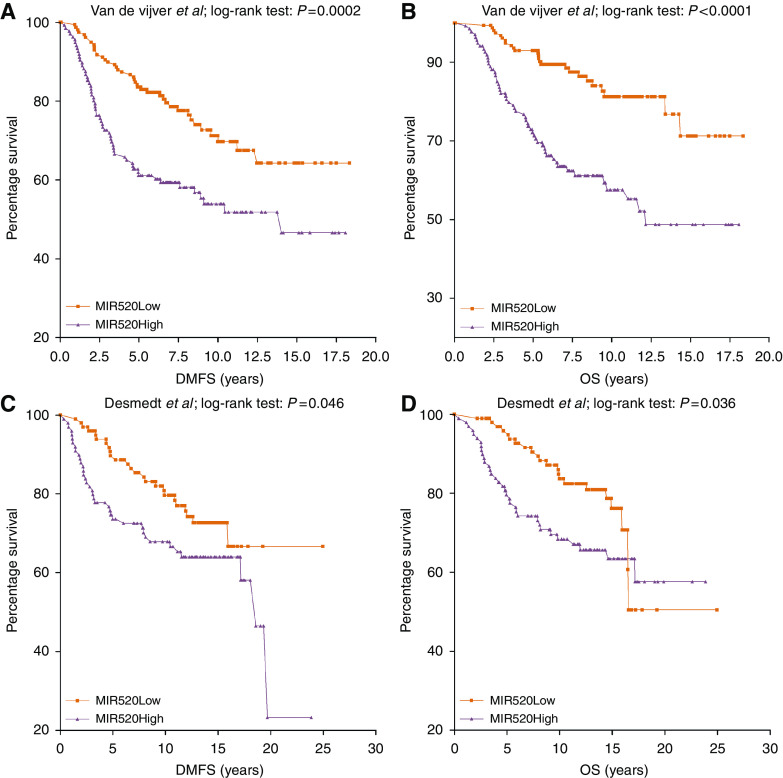
Kaplan–Meier survival analysis of miR-520a-5p target gene expression in breast cancer with distant metastasis-free (**A** and **C**) and overall survival (**B** and **D**) as outcome. Kaplan–Meier curves are shown for the data sets of van de Vijver *et al* (2002) (**A** and **B**) and of Desmedt *et al* (2007) (**C** and **D**).

To investigate the potential prognostic relevance of miR-29a, miR-30b, miR-342-3p and miR-520a-5p in breast cancer, we performed a Cox regression analysis for seven publicly available gene expression data sets for which information regarding DMFS, RFS or OS was available using an indirect approach. First, we calculated for each sample an miRNA target gene expression score by subtracting the average relative gene expression value of the negatively correlated miRNA target genes from the average relative gene expression value of the positively correlated miRNA target genes (Figure 3). The resulting score was standardised (median=0, s.d.=1) to allow for comparison across different data sets. The miRNA target gene expression scores calculated as such were all strongly correlated with the miRNA expression values, thereby validating the score (range Spearman correlation coefficients: 0.70–0.76; *P*<0.0001). The association between outcome and the miRNA target gene expression score was evaluated using Kaplan–Meier and Cox regression analysis (Table 7). Overall, we observed significant associations between miRNA target gene expression and patient outcome (DMFS, RFS and OS) for miR-29a, miR-30b and miR-520a-5p, but not for miR-342-3p. In particular, high levels of miR-520a-5p target genes correlated with shorter DMFS, RFS and OS in, respectively, 3 out of 4, 3 out of 5 and 3 out of 3 data sets and performed best in a Cox regression analysis.

### Expression of miRNA processing genes in breast cancer

Recently, Cheng *et al* (2009) reported that the post-transcriptional regulation of miRNA expression may be important for the regulatory effect of miRNAs on their targets (Cheng *et al*, 2009). We therefore examined whether miRNA processing genes (*trbp2*, *dicer*, *ago1*, *ago2* and *drosha*) are differentially expressed between IBC and non-IBC. We observed that among the miRNA processing genes, *ago2* was significantly upregulated and *dicer* significantly downregulated in IBC (*N*=20) compared with non-IBC samples (*N*=24), with P-values of 0.002 and 0.004 (false discovery rate <0.1), respectively. We further examined whether this altered expression of *ago2* and *dicer* in IBC when compared with non-IBC is also reflected in altered regulatory effects of miRNAs on their targets. We calculated an RE score for each of 153 representative miRNA families from TargetScan 5.1 in all 44 samples using a strategy adapted from Cheng *et al* (2009). Then, we compared RE scores between IBC and non-IBC samples. Of 153 miRNA families, 104 (68%) showed higher RE scores in IBC and 49 (32%) showed higher RE scores in non-IBC. Using the significance analysis of microarrays method, we observed 20 significant RE-changing miRNAs (miRNAs that show different regulatory effects between IBC and non-IBC).

## Discussion

MiRNAs are a recently discovered class of small regulatory RNAs, which influence the stability and translational efficiency of target mRNAs (Verghese *et al*, 2008). In this study, we examined the expression of 384 miRNAs in 70 breast cancer samples to look for miRNAs, of which expression levels are significantly different in IBC compared with non-IBC. The spectrum of expressed miRNAs mostly varied according to steroid hormone receptor expression and histological tumour grade and not according to tumour subtype (IBC *vs* non-IBC). However, we did identify a set of 13 miRNAs for which expression levels were able to correctly predict the nature of the sample analysed (IBC *vs* non-IBC). Six of them, miR-335, miR-337-5p, miR-451, miR-486-3p, miR-520a-5p and miR-548d-5p, were upregulated in IBC and the remaining seven, miR-15a, miR-24, miR-29a, miR-30b, miR-320, miR-342-5p and miR-342-3p, were downregulated in IBC. The observation that few miRNAs are specifically associated with IBC does not support the hypothesis of a prominent role for altered miRNA expression in the phenotype of IBC. However, miRNA expression levels are not necessarily representative of the regulatory ability of an miRNA. Cheng *et al* (2009) recently reported that the inhibitory effects of miRNAs on their targets differs between two breast cancer subtypes (ER^−^ and ER^+^) due to a differential expression of several key miRNA processing genes and not due to differences in miRNA expression levels (Cheng *et al*, 2009). We therefore examined the expression levels of genes in the miRNA biogenesis pathway in samples from IBC and non-IBC. We observed significantly lower expression levels for *dicer*, a ribonuclease, which cleaves the pre-miRNA into a single-stranded mature miRNA and significantly higher expression levels for *ago2*, the catalytic endonuclease of the RNA-induced silencing complex in IBC when compared with non-IBC. The latter observation may suggest higher RNA-induced silencing complex activities, and therefore may further suggest that miRNAs regulate target gene expression in IBC with higher efficiency. Indeed, miRNAs seem to have a higher regulatory effect (reflected by the RE score) in IBC samples than in non-IBC samples. It has been previously observed that low *dicer* expression and high *ago2* expression in breast cancer is associated with the more aggressive basal-like, HER2+ and luminal B subtypes (Blenkiron *et al*, 2007), which are known to be overrepresented in IBC (Van Laere *et al*, 2006). Moreover, decreased *dicer* expression has been reported to be associated with poor clinical outcome in breast, ovarian and lung cancer (Karube *et al*, 2005; Merritt *et al*, 2008; Grelier *et al*, 2009).

At present, the lack of knowledge about *bona fide* miRNA target genes hampers a full understanding on the biological functions deregulated by aberrant miRNA expression. To overcome this limitation, we adopted the strategy recently described by Wang *et al* (2009) for the miRNAs of which expression differed between IBC and non-IBC (Wang *et al*, 2009). First, we used a whole genome approach to identify thousands of highly correlated miRNA–mRNA pairs. We identified a large number of both negative and positive correlations. The detection of a positive correlation between miRNA and mRNA levels suggests a positive regulatory role for miRNAs, as has been reported for some genes (Vasudevan *et al*, 2007). Second, we made use of a computational approach to search for predicted miRNA targets. Concordant genes are direct miRNA targets that are tightly correlated with fluctuations in miRNA expression. We observed significant concordance between miRNA-correlated genes and miRNA-predicted targets in only 4 of 13 miRNA sets (miR-29a, miR-30b, miR-342-3p and miR-520a-5p). Thus, we did not see any evidence of concordance in a majority of miRNA sets. Generally, miRNAs are believed to bind 3′ untranslated region of a target gene and regulate gene expression at protein level (Wang *et al*, 2009). Therefore, miRNA targets themselves may not demonstrate noticeable change at the mRNA level. However, this can also be explained by discordance in changes of expression between the key miRNA processing genes. For miR-29a, miR-30b, miR-342-3p and miR-520a-5p target genes, we were able to detect a variety of biological processes that may indicate function of those miRNAs. MiR-29a target genes were mainly related to DNA methyltransferase activity. Indeed, expression of the miR-29 family has been shown to target DNA methyltransferase 3A and 3B expression in lung cancer tissues (Fabbri *et al*, 2007) and in acute myeloid leukaemia (Garzon *et al*, 2009), inducing global hypomethylation. For miR-30b, we observed target genes related to insulin receptor signalling. Recently, it has been reported that another member of the miRNA-30 family, miR-30d, is upregulated by glucose and increases insulin gene expression (Tang *et al*, 2009). MiR-342-3p and miR-520a-5p target genes proved to be involved in cell proliferation.

As IBC is regarded as a model for breast cancer aggressiveness, we were interested in determining whether the IBC-specific miRNAs were associated with a dismal prognosis in non-IBC. Given the limited number of our clinical samples, we used an indirect approach and correlated miRNA target gene expression with patients’ outcome using publicly available information from microarrays studies, which could be extracted from the gene expression omnibus database. This analysis demonstrated a marked association of miR-520a-5p target gene expression with a shorter DMFS, RFS or OS in most breast cancer data sets that were investigated. This result demonstrates a possible role of miR-520a-5p as a prognostic factor in breast cancer. Although we acknowledge the limitations of using an indirect approach, these kinds of analyses may help to guide future large-scale studies on the prognostic value of miRNAs in breast cancer.

In conclusion, this study, to the best of our knowledge, represents the first integrated analysis of miRNA and mRNA expression in IBC. We identified a number of miRNAs that are differentially expressed between IBC and non-IBC. Furthermore, we reported altered expression of miRNA processing genes in IBC when compared with non-IBC. For four of the IBC-related miRNAs, we were able to detect a variety of biological processes that may indicate function of these miRNAs and to indicate their potential association with prognosis in breast cancer based on the expression levels of their target genes.

## Figures and Tables

**Table 1:** Tumour characteristics

**Clinicopathological features**	**IBC (*N*=20)**	**Non-IBC (*N*=50)**	***P*-value**
*Patients’ ages (years)*			
Mean	60	59	0.692
Range	45–79	30–89	
			
*Tumour stage*			
I	0 (0%)	21 (42%)	
II	0 (0%)	15 (30%)	<0.001
III	10 (50%)	11 (22%)	
IV	10 (50%)	3 (6%)	
			
*Histological tumour grade* a			
Well	0 (0%)	8 (16%)	
Moderate	9 (45%)	23 (46%)	0.125
Poor	11 (55%)	19 (38%)	
			
*Oestrogen receptor*			
Positive	16 (80%)	35 (70%)	0.395
Negative	4 (20%)	15 (30%)	
			
*HER2 amplification*			
Positive	8 (40%)	9 (18%)	0.052
Negative	12 (60%)	41 (82%)	

Abbreviations: HER2=human epidermal growth factor receptor 2; IBC=inflammatory breast cancer.

a Nottingham histological grade (Elston, 1984).

**Table 2:** Association of global miRNA expression with clinicopathological factors

**Factor**	**50 miRNAs with greatest standard variation**
T status	*P*=0.646
N status	*P*=0.092
M status	*P*=0.141
Stage	*P*=0.332
Histological grade	*P*=0.002
ER expression	*P*=0.001
HER2 amplification	*P*=0.274
Tumour subtype (IBC or non-IBC)	*P*=0.219

Abbreviations: ER=oestrogen receptor; HER2=human epidermal growth factor receptor 2; IBC = inflammatory breast cancer; miRNA, microRNA.

**Table 3:** Association of miRNA expression with clinicopathological factors

**Factor**	**Upregulated**	**Downregulated**
T1/2 *vs* T3/4	—	miR-337-5p; miR-369-5p; miR-455-5p
N+ *vs* N0	miR-216a; miR-155; miR-891a	miR-31; miR-138; miR-140-3p; miR-140-5p; miR-146a; miR-150; miR-186; miR-330-5p; miR-363; miR-374a; miR-450a, miR-489; miR-542-3p
M^+^ *vs* M0	miR-96; miR-128; miR-130b; miR-216b; miR-372; miR-423-5p; miR-425; miR-431	miR-150; miR-202; miR-223; miR-548d-5p; miR-589; miR-618; miR-629; miR-636; miR-506
Stage I/II *vs* Stage III/IV	miR-302b; miR-576-5p; miR-642	miR-30c; miR-130a; miR-337-5p
Grade 3 *vs* Grade 1/2	miR-9; miR-18a; miR-25; miR-28-5p; miR-98; miR-106b; miR-128; miR-130b; miR-139-3p; miR-181a; miR-200c; miR-203; miR-210; miR-324-5p; miR-340; miR-362-3p; miR-455-3p; miR-455-5p; miR-483-5p; miR-486-3p; miR-486-5p; miR-551b	let-7c; miR-16; miR-18b; miR-126; miR-133a; miR-139-5p; miR-140-3p; miR-140-5p; miR-181c; miR-204; miR-208b; miR-218; miR-301b; miR-502-3p; miR-505; miR-509-5p; miR-548d-3p; miR-576-3p; miR-579; miR-636; miR-376c
ER^+^ *vs* ER^−^	miR-10a; miR-10b; miR-18b; miR-29c; miR-145; miR-149; miR-193b; miR-199a-5p; miR-328; miR-155; miR-342-3p; miR-422a; miR-503; miR-505; miR-511	miR-18a; miR-19a; miR-34c-5p; miR-106a; miR-139-3p; miR-142-3p; miR-142-5p; miR-146a; miR-146b-5p; miR-154; miR-199b-5p; miR-222; miR-224; miR-299-5p; miR-362-3p; miR-409-5p; miR-433; miR-486-5p; miR-499-5p; miR-532-3p; miR-532-5p; miR-615-3p; miR-874
HER2^+^ *vs* HER2^−^	miR-33b; miR-184; miR-216b; miR-331-3p; miR-425; miR-520a-3p	let-7c; miR-26b; miR-29a; miR-29c; miR-30c; miR-101; miR-130a; miR-148a; miR-195; miR-205; miR-324-3p; miR-455-3p; miR-455-5p; miR-485-3p
IBC *vs* non-IBC	miR-335; miR-451; miR-520a-5p; miR-548a-5p; miR-337-5p; miR-486-3p	miR-15a; miR-24; miR-29a; miR-30b; miR-320; miR-342-3p; miR-342-5p

Abbreviations: ER=oestrogen receptor; HER2=human epidermal growth factor receptor 2; IBC=inflammatory breast cancer; miRNA=microRNA.

**Table 4:** Correlations between miRNAs and mRNAs in breast cancer (*N*=44)

	**Total number of correlations**	**Negative correlation**	**Positive correlation**	**GSEA**^a^ ***P*-value**	**Intersect with TargetScan**	**GSEA**^b^ ***P*-value**
miR-15a	98	35	63	<0.001	9 (9%)	0.652
miR-24	8	2	6	0.003	0 (0%)	NA
miR-29a	2925	1300	1625	<0.001	156 (5%)	0.0001
miR-30b	1962	658	1304	<0.001	199 (10%)	0.003
miR-320	201	81	120	<0.001	13 (6%)	0.697
miR-335	575	181	394	0.007	8 (1%)	0.307
miR-342-5p	3029	1146	1883	<0.001	13 (<1%)	0.416
miR-342-3p	3580	1296	2284	<0.001	31 (<1%)	0.0001
miR-337-5p	1650	712	938	0.03	1 (<1%)	0.528
miR-451	322	186	136	0.001	2 (<1%)	0.256
miR-486-3p	95	46	49	NS	NA	NA
miR-520a-5p	1011	586	425	0.03	17 (2%)	0.028
miR-548d-5p	1839	783	1056	0.02	111 (6%)	0.554

Abbreviations: GSEA=gene set enrichment analysis; IBC=inflammatory breast cancer; mRNA=messenger RNA; miRNA=microRNA; NA=not available; NS=non-significant.

a Gene set enrichment analysis of miRNA-correlated genes using a list of all genes that are differentially expressed in IBC and non-IBC as a reference set.

b Gene set enrichment analysis of predicted target genes by TargetScan using the miRNA-correlated genes as a reference set.

**Table 5:** Gene symbols for direct miRNA target genes

**miRNA**	**Direct target genes**
miR-29a	MGC21874, BRWD1, MIER3, HBP1, THSD4, FAM116A, USP37, AFF4, ZBTB40, UBTF, SP1, PALM, DNAL1, MTX3, MAP2K4, KIAA0831, SLC39A9, JMJD2B, LCORL, KLHL9, SESTD1, RERE, C5orf24, USP34, NEBL, PIAS4, NKTR, JARID1A, AMFR, ARPP-19, CCNL2, CAPN7, BSDC1, TTC30B, ELF2, RAP1GDS1, LNPEP, GCC2, ERLIN2, PAN2, NCOR2, GNG12, EML5, STX17, CNOT8, MSL-1, RHBDD1, tcag7.1228, ATRN, FAM168B, PRPF40A, DICER1, KLHL28, PAIP2, BTBD7, ARFGEF2, LOC153364, SR140, BTG2, C19orf6, RIT1, SLC7A6, EML4, TTYH2, TNFAIP3, FBXL11, NANP, NUP160, TRAM2, NANOS1, IFI30, CDK6, MLXIP, MYCN, MAPRE2, MAP4K4, CHFR, LUZP1, SLC36A1, TRIB2, FSTL1, SLC16A14, CBX2, MEST, NCOA3, CDCA4, DIO2, DNAJB11, DNMT3A, NUFIP2, IMPDH1, INSIG1, OSBPL3, MAPRE1, PHACTR2, TFEC, EIF4E2, ABCE1, TSPAN14, SERPINH1, B3GNT5, PLXNA1, RPS6KA3, GNB4, DTX4, HMGCS1, DEF8, KIAA1128, COMMD2, SLC2A3, MYBL2, TET3, CCNJ, TMEM65, DPP4, JOSD1, DNMT3B, TAF11, CYCS, TBC1D7, CHIC2
miR-30b	NRIP1, RAPGEF6, BECN1, TRPS1, HIPK2, NF1, VAPA, BRWD1, SLC1A2, IGF1R, USP37, CSNK1G1, MIER3, TSGA14, KCTD3, KIAA1712, AFF3, ANKHD1, ZNF770, C2orf55, CHD1, SLC4A7, GIGYF2, CPEB4, IRS1, CCNT2, MKL2, CSAD, PAPOLA, VAV3, LCORL, C15orf29, DCTN4, SCAMP1, NEK4, MTX3, PHF13, ELOVL5, CCPG1, MAGI2, AFF4, LASS6, GZF1, CPEB2, FLJ40142, HNRNPA3, BCL2L11, ZBTB40, ABHD2, EPC2, ZNF148, SNX1, TNKS, ATRN, PHC3, TTC8, REV1, MNT, ARID4A, MGC21874, PPP1R9A, RBM12, FBXL17, ZDHHC17, KIAA1632, USP47, TMEM106B, PCGF3, KIAA0999, MSI2, CLCC1, MBD6, SIRT1, PSME3, PIP4K2B, CXorf39, KIF3A, TTC39A, IRS2, FAM110B, BCL6, PNKD, PAWR, PPAPDC1B, BCL2, MARCKS, SH2B3, ZNRF1, WDR26, CHFR, IGF2R, LYCAT, CAMK2D, ARL4C, CCNK, IFNAR2, NT5E, SLC36A1, FBXO45, MFHAS1, PHACTR2, TET3, EML4, SMAP1, FAM152A, CALU, TRAM2, ITGA5, KPNA3, GRK6, LYN, SURF4, RASSF4, RAP2B, NRBF2, NCOA3, FAM43A, LMBR1 L, MAP4K4, SUV39H2, MYO5A, KLHL20, ME1, DNMT3A, MYBL2, LCP1, SMAD1, KIAA1949, PHTF2, SPTLC2, CHST2, QKI, SOCS1, SEC24A, FLJ36031, CLN8, CAPZA1, DBF4, KLF11, IDH1
miR-342-3p	MRFAP1, CA12, ACVR2B, PCGF3, MSI2, LARP4, ZAK, AAMP, HIP1, NBEA, UQCC, PTRF, SHE, ID4, SSR1, MEX3A, PDGFRA, NCOA7, CDK6
miR-520a-5p	PPP1R9B, SMEK1, HEG1, SLC25A13, TMPO, ABCE1, KPNA1, PTP4A2, BAG1, ARHGEF12, NOVA1, PDPK1, LZTFL1

Abbreviation: miRNA=microRNA.

**Table 6:** Association of direct miRNA target genes with biological processes

**miRNA**	**ID**	**Term**	***P*-value**
miR-29a	GO:0003886	DNA (cytosine-5-) methyltransferase activity	0.0001
	GO:0010468	Regulation of gene expression	0.0001
	GO:0009008	DNA methyltransferase activity	0.0003
	GO:0043414	Biopolymer methylation	0.0010
	GO:0032259	Methylation	0.0016
	GO:0009889	Regulation of biosynthetic process	0.0020
	GO:0050794	Regulation of cellular process	0.0025
	GO:0010467	Gene expression	0.0026
	GO:0040029	Regulation of gene expression, epigenetic	0.0027
	GO:0006305	DNA alkylation	0.0034
miR-30b	GO:0043548	Phosphoinositide 3-kinase binding	<0.0001
	GO:0008286	Insulin receptor signalling pathway	0.0004
	GO:0005010	Insulin-like growth factor receptor activity	0.0005
	GO:0000122	Negative regulation of transcription from RNA pol II promoter	0.0007
	GO:0019899	Enzyme binding	0.0009
	GO:0043434	Response to peptide hormone stimulus	0.0009
	GO:0009725	Response to hormone stimulus	0.0009
	GO:0009719	Response to endogenous stimulus	0.0010
	GO:0045892	Negative regulation of transcription, DNA dependent	0.0011
	GO:0005158	Insulin receptor binding	0.0011
miR-342-3p	GO:0008284	Positive regulation of cell proliferation	<0.0001
	GO:0048146	Positive regulation of fibroblast proliferation	<0.0001
	GO:0048144	Fibroblast proliferation	0.0001
	GO:0048145	Regulation of fibroblast proliferation	0.0001
	GO:0048522	Positive regulation of cellular process	0.0001
	GO:0045667	Regulation of osteoblast differentiation	0.0001
	GO:0042063	Gliogenesis	0.0002
	GO:0048518	Positive regulation of biological process	0.0002
	GO:0001649	Osteoblast differentiation	0.0003
	GO:0008283	Cell proliferation	0.0004
miR-520a-5p	GO:0001560	Regulation of cell growth by extracellular stimulus	0.0011
	GO:0000164	Protein phosphatase type 1 complex	0.0012
	GO:0015810	Aspartate transport	0.0022
	GO:0043490	Malate–aspartate shuttle	0.0022
	GO:0004727	Prenylated protein tyrosine phosphatase activity	0.0025
	GO:0008157	Protein phosphatase 1 binding	0.0025
	GO:0015183	L-aspartate transmembrane transporter activity	0.0025
	GO:0044453	Nuclear membrane part	0.0033
	GO:0005521	Lamin binding	0.0063
	GO:0031965	Nuclear membrane	0.0068

Abbreviation: miRNA=microRNA.

**Table 7:** Association of direct miRNA target genes with prognosis in breast cancer

	**DMFS**			**RFS**			**OS**		
**miRNA**	**Data set**	**HR**	***P*-value**	**Data set**	**HR**	***P*-value**	**Data set**	**HR**	***P*-value**
miR-29a	Desmedt *et al* (2007)	0.836	0.156	Desmedt *et al* (2007)	0.893	0.276	Desmedt *et al* (2007)	0.800	0.092
	Schmidt *et al* (2008)	0.738	0.038	Loi *et al* (2008)	0.549	0.028	Pawitan *et al* (2005)	0.564	0.001
	Loi *et al* (2008)	0.487	0.021	Wang *et al* (2007)	0.874	0.159	van de Vijver *et al* (2002)	0.595	0.0001
	van de Vijver *et al* (2002)	0.736	0.001	Ivshina *et al* (2006)	0.846	0.127	—	—	—
	—	—	—	Pawitan *et al* (2005)	0.617	0.001	—	—	—
	Total (*N*=770)	0.760	0.0001	Total (*N*=1009)	0.815	0.001	Total (*N*=652)	0.652	0.0001
miR-30b	Desmedt *et al* (2007)	0.789	0.066	Desmedt *et al* (2007)	0.912	0.374	Desmedt *et al* (2007)	0.766	0.049
	Schmidt *et al* (2008)	0.789	0.083	Loi *et al* (2008)	0.560	0.022	Pawitan *et al* (2005)	0.575	0.002
	Loi *et al* (2008)	0.535	0.030	Wang *et al* (2007)	0.919	0.378	van de Vijver *et al* (2002)	0.510	0.0001
	van de Vijver *et al* (2002)	0.679	0.0001	Ivshina *et al* (2006)	0.949	0.637	—	—	—
	—	—	—	Pawitan *et al* (2005)	0.630	0.002	—	—	—
	Total (*N*=770)	0.745	0.0001	Total (*N*=1009)	0.837	0.004	Total (*N*=652)	0.600	0.0001
miR-342-3p	Desmedt *et al* (2007)	0.944	0.661	Desmedt *et al* (2007)	1.086	0.456	Desmedt *et al* (2007)	0.936	0.622
	Schmidt *et al* (2008)	1.003	0.985	Loi *et al* (2008)	1.035	0.911	Pawitan *et al* (2005)	0.978	0.911
	Loi *et al* (2008)	0.908	0.773	Wang *et al* (2007)	0.919	0.391	van de Vijver *et al* (2002)	0.787	0.027
	van de Vijver *et al* (2002)	0.977	0.830	Ivshina *et al* (2006)	1.102	0.409	—	—	—
	—	—	—	Pawitan *et al* (2005)	0.983	0.918	—	—	—
	Total (*N*=770)	0.979	0.763	Total (*N*=1009)	0.993	0.918	Total (*N*=652)	0.870	0.078
miR-520a-5p	Desmedt *et al* (2007)	1.452	0.005	Desmedt *et al* (2007)	1.263	0.027	Desmedt *et al* (2007)	1.471	0.006
	Schmidt *et al* (2008)	1.285	0.083	Loi *et al* (2008)	2.216	0.009	Pawitan *et al* (2005)	2.080	0.0001
	Loi *et al* (2008)	2.651	0.006	Wang *et al* (2007)	1.204	0.062	van de Vijver *et al* (2002)	1.828	0.0001
	van de Vijver *et al* (2002)	1.488	0.0001	Ivshina *et al* (2006)	1.163	0.169	—	—	—
	—	—	—	Pawitan *et al* (2005)	1.855	0.0001	—	—	—
	Total (*N*=770)	1.452	0.0001	Total (*N*=1009)	1.344	0.0001	Total (*N*=652)	1.762	0.0001

Abbreviations: DMFS=distant metastases-free survival; HR=hazard ratio; miRNA=micro RNA; OS=overall survival; RFS=relapse-free survival.
